# Nasal Dermoids in Children: Factors Influencing the Distant Result

**DOI:** 10.1007/s12070-021-02568-y

**Published:** 2021-04-22

**Authors:** Michal Kotowski, Paulina Adamczyk, Jaroslaw Szydlowski

**Affiliations:** Department of Pediatric Otolaryngology, Poznan University of Medical Sciences, 27/33 Szpitalna Street, 60-572 Poznan, Poland

**Keywords:** Nasal dermoid, Intracranial extension, Anterior skull base, Children

## Abstract

The aim of the study was to present a single institution’s treatment strategy for nasal dermoids and to identify factors influencing distant results. The study covered 24 surgically treated pediatric patients with nasal dermoids (NDs). The medical data concerning demographics, preoperative local inflammations and surgical procedures, form of the abnormality, imaging, surgical techniques, and a role of osteotomies and reconstructions were analyzed. The recurrence rates and distant aesthetic outcomes were assessed. The surgical approach included vertical incision in 21 patients, the external rhinoplasty approach in 2 cases, and bicoronal incision in 1 child. The intracranial extension was confirmed in 6 patients. Seven out of 8 cases with preoperative local inflammations and 3 out of 4 with secondary fistulization were < 4 years old. Nine patients required osteotomies. Three children required reconstruction of the nasal skeleton. None of the distant cosmetic results was described as hideous or unsatisfactory. The incidence of local inflammatory complications is unrelated to the age of the patients. The distant aesthetic result depends on both the initial extent of the disease and preoperative local conditions or interventions. Prompt surgical intervention is highly recommended.

## Introduction

Midline congenital nasal masses are rare developmental abnormalities. The incidence is estimated at 1:20,000–40,000 live births [1]. The group includes mainly dermoids (60%), nasal gliomas and encephaloceles [1, 2]. Epidermoid cyst, teratoma, vascular anomaly, neurofibroma, and lipoma must be also considered in the differential diagnosis.

Nasal dermoids (NDs) comprise ecto and mesodermal components. They are typically lined by squamous epithelium, and natural dermal appendages as hair and sebaceous glands are present. Embriologically NDs are the consequence of the improper process of the dural diverticulum from the skin between the 4th and 8th week of gestation described in previous publications [3, 4]. Inconsistent reports on these lesions' familial basis have been published [2, 5–8].

Various craniofacial abnormalities may accompany them in 0–41%—craniosynostosis, hemifacial microsomia, lacrimal duct cysts, cleft lips/palates, pinna deformities, hydrocephalus, and hypertelorism [8–11].

A midline mass has been thought to have a form of the cyst or sinus with its skin opening anywhere from base of columella to glabellar region. A hair visible in the ostium is considered to be pathognomonic for a nasal dermoid. The significant features in the differential diagnosis are non-compressible mass with no transillumination and reported intermittent discharge of sebaceous material or pus from the skin ostium. An observation of potential enlargement during cry or Furstenberg’s test is typically negative. The intracranial extension is well documented in the literature and estimated 4–57% [7–10, 12–14].

Nasal dermoids may result in a variety of local and intracranial complications. Local consequences include inflammation, abscess formation, granulation, secondary fistula formation, in some cases initiated by an external trauma [10]. Intracranial complications cover meningitis, cerebral abscess, periorbital cellulitis, cavernous sinus thrombosis [1, 5, 9, 10, 15]. The total resection is the treatment of choice. Recurrence rates are estimated 5.5–12% [10, 16]. The risk of recurrence increases dramatically when removed incompletely, reaching 50–100% [17]. Due to the rarity of these lesions, large series of pediatric patients are uncommon in the literature.

The aim of the study was to present a single institution’s treatment strategy for nasal dermoids and to identify factors influencing distant results. Moreover, it was to discuss viable perioperative considerations concerning the age of the surgery, radiological imaging, role of osteotomies and reconstructions, surgical approaches and cosmetic results.

## Material and Methods

### Study Group

The conducted research included 24 pediatric patients with NDs, aged from 6 months to 17 years, treated surgically at the Department of Pediatric Otolaryngology, Poznan University of Medical Sciences, Poland, from 2014 to 2020. The preoperative radiological imaging (CT, MR, or both) was performed in all patients.

### Surgical Procedure

Surgical procedures included checking the canal patency, length, and direction with a lacrimal probe in case of the skin orifice presence. If possible, the violet dye was injected into the sinus lumen for better visualization during the dissection. According to the form of the abnormality a vertical incision, an external rhinoplasty, bicoronal or endoscopic-assisted approaches were implemented. In the case of secondary fistulization, the approach was customized. The dissection was focused on providing *en bloc* total resection. In the case of intraosseous and intracranial lesions, osteotomies were performed, and the drill was used if necessary. Intracranial lesions required following the tract to its blind end or dissection from the dura.

### Medical Data

The medical data concerning demographics, symptoms, episodes of local inflammations, previous surgical procedures, imaging studies, surgical techniques, and a role of osteotomies and reconstructions were analyzed. Based on the localization and the presence of intracranial extension, which was verified intraoperatively, the patients’ cohort was divided into subgroups according to the classification proposed by Hartley et al. [12]: superficial, intraosseous, intracranial-extradural, and intracranial-intradural. During the follow-up period the recurrence rates and distant aesthetic outcomes were assessed. On the basis of parents’/guardians’ opinion, the aesthetic result was evaluated using a five-degree scale (hideous, unsatisfactory, satisfactory, good, excellent).

## Results

**Fig. 1:**
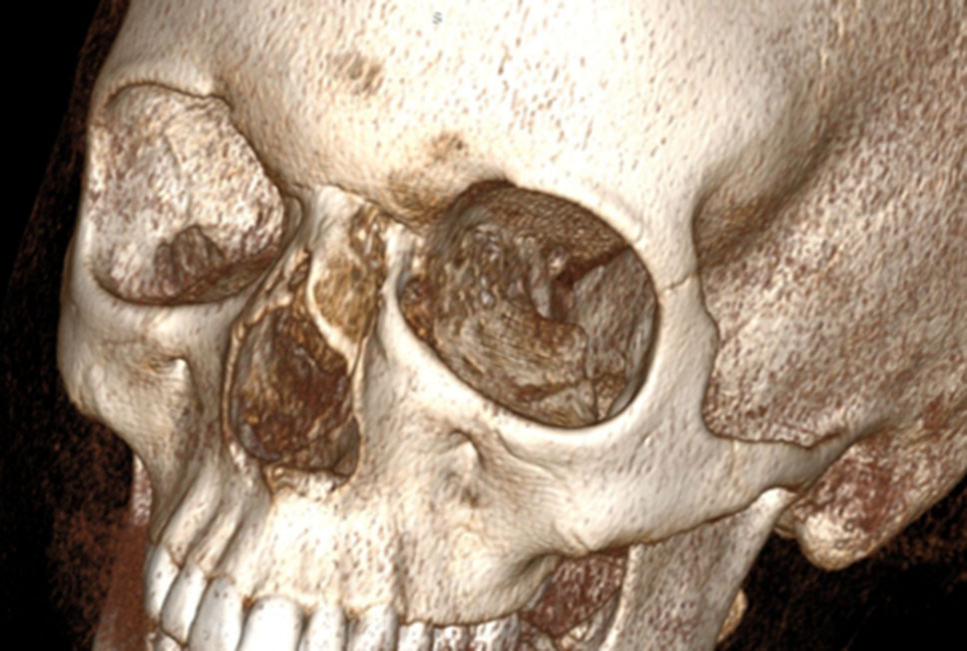
Deformation and atrophy of the nasal bones in 12-year-old girl with nasal dermoid—CT scan & 3D reconstruction

The study group consisted of 14 male and 10 female patients. The average time of surgery was 5 years and 4 months (range 6 months–17 years). An anamnesis revealed 8 children suffering from recurrent local inflammations prior to the surgery. Five of them reported different surgical interventions (incisions, drainage, laser excision, incomplete removal etc.) at other institutions. The significant deformity of the external nose was observed in 6 cases. (Fig. 1). Four of them manifested secondary fistula with the granulation tissue and skin damage. All children underwent preoperative radiological imaging: computed tomography in 11 cases, magnetic resonance in 6 patients. Both imaging tools were used in 7 cases. According to Hartley’s classification, 11 children presented superficial cyst or sinus, and in 7 cases, the intraosseous lesion was revealed. The intracranial extension was confirmed in 6 patients (25%), and these were classified as an intracranial extradural form of the abnormality. In that subgroup, one child manifested as extremely rare case of philtrum sinus with the intracartilaginous and intraosseous tract to the anterior fossa dura. No accompanying craniofacial abnormalities were revealed.

The surgical approach via vertical incision was used in 21 patients, the external rhinoplasty approach with endoscopic resection was performed in 2 cases, and one child with an abscess of the frontal region required bicoronal incision. An intraoperative CSF leak occurred in 2 cases (8,3%) and was simultaneously repaired using a fibrin sealant patch. Nine patients (37.5%) required osteotomies during the surgical procedure. Three children required simultaneous reconstruction of the nasal skeleton due to the significant deformity (cartilage costal graft, fat graft, and bone chips were used in one case, respectively).

The confirmation of nasal dermoid based on a routine histopathology in each case. The follow-up period ranged from 6 months to 5 years. The parents’ or guardians’ evaluation of the distant cosmetic result was collected 12 months after the surgery in 21 cases. None was described as hideous or unsatisfactory. Seven of them were regarded as satisfactory, good in 11 cases, and excellent in 3 patients. The mean age in these subgroups was 5.1/4.9/4.8 years old, respectively. All the patients with “satisfactory” result reported the medical history of recurrent local inflammations, surgical interventions or recurrence of the disease. The recurrence rate was 8.3% (2 patients). Both of them were identified after 12–18 months after the initial surgery and successfully reoperated. The detailed results of the study are summarized in Table 1.

**Table 1:** Detailed results in the study group

	Patient	Sex	Age	Form	Imaging	Surgical approach	Cosmetic result
1	PR	F	7	S	CT	VI	G
2	MN	M	3	S	CT	VI	E
3	FH	M	1.5	S	CT	VI	G
4	LS	M	2.5	S	MRI	VI	S^R^
5	AB	F	2.5	IO	MR	VI	G
6	WR	F	12.5	IO^1,2^	CT	VI^3,4a^	S^6^
7	AS	F	0.5	IC^1,2^	CT + MRI	VI ^3^	S^5,6,R^
8	TL	M	4	S	CT	ER	E
9	MM	M	7	S	CT	VI	G^5^
10	MM	M	3	IO^1,2^	CT	VI ^3^	S^5,6^
11	MB	F	2	S	MRI	VI	G^6^
12	WL	F	3.5	S	CT	VI	S^6^
13	AP	M	0.75	IC	CT + MRI	VI^3^	G
14	HK	M	15	S	CT	VI	G^5^
15	HL	F	3	IC^1,2^	CT + MRI	VI^4b^	G^5,6^
16	BK	M	7.5	IC*	CT + MRI	ER^CSF^	E
17	ET	F	10.5	IO^1^	MRI	VI^3^	S^5^
18	WO	F	3	IO	CT + MRI	VI^3^	S^6^
19	AS	M	1.25	IC	MRI	VI^3,CSF^	G
20	KW	M	12.5	S	CT	VI	G
21	AM	M	1.5	IC^**^	CT + MRI	BC^4c^	G ^6^
22	KD	F	2.5	S	MRI	VI	N/A
23	BZ	M	4.5	IO	CT	VI^3^	N/A
24	AB	M	17	IO^1^	CT + MRI	VI^3^	N/A

Form: *S* superficial, *IO* intraosseous, *IC* intracranial, ^1^significant deformity of the external nose, ^2^secondary fistula with the granulation tissue and skin damage, *philtrum sinus with intracranial extension; **frontal abscess

Imaging: *CT* computed tomography, *MRI* magnetic resonance imaging;

Surgical approach: *VI* vertical incision, *ER* external rhinoplasty with total endoscopic resection, *BC* bicoronal approach, ^3^osteotomy, ^4^reconstruction (^a^cartilage costal graft, ^b^fat tissue, ^c^bony chips), *CSF* cerebrospinal fluid leak;

Cosmetic result: *S* satisfactory, *G* good, *E* excellent, *N/A* non-applicable (follow-up < 12 months), ^5^previous surgical procedures or local trauma in anamnesis, ^6^recurrent local inflammations prior to the surgery, ^R^recurrence of the disease

## Discussion

### Epidemiology

Nasal dermoids are rare congenital abnormalities. Their incidence is equivalent to approximately 10 new patients per year in Poland and 100 new cases each year in the USA. This is clearly reflected in the literature, which is dominated by case reports and short series. The largest series was published by GOSH group as they collected 103 cases in 12 years [12] and the Boston Group–96 patients in 45 years [16]. Even most of representative centers of the highest referral level cover 2–2.5 cases per year [10, 11, 16, 18, 19]. Slight male predominance was reported in the literature and was observed in our study (M:F = 1.4:1). [7, 8, 10, 12, 14].

### Classification

Depending on the disease’s location and extent, nasal dermoids were divided by Hartley et al. into four groups: superficial, intra-osseous, intracranial extradural, and intracranial intradural [12]. Our case of philtrum sinus with intracranial extension was classified as intracranial-extradural, but it stimulates to revise the current classification. The literature review, which was performed provided 3 previously reported cases of the middle upper lip fistula with the intracranial extension [20]. Basing on the undeniable embryological and clinical similarity, the additional forms of this abnormality covering superficial, intracartilaginous, intraosseous, and intracranial manifestations of middle upper lip fistulas and sinuses should be added [21, 22].

### Age for Surgery

**Fig. 2:**
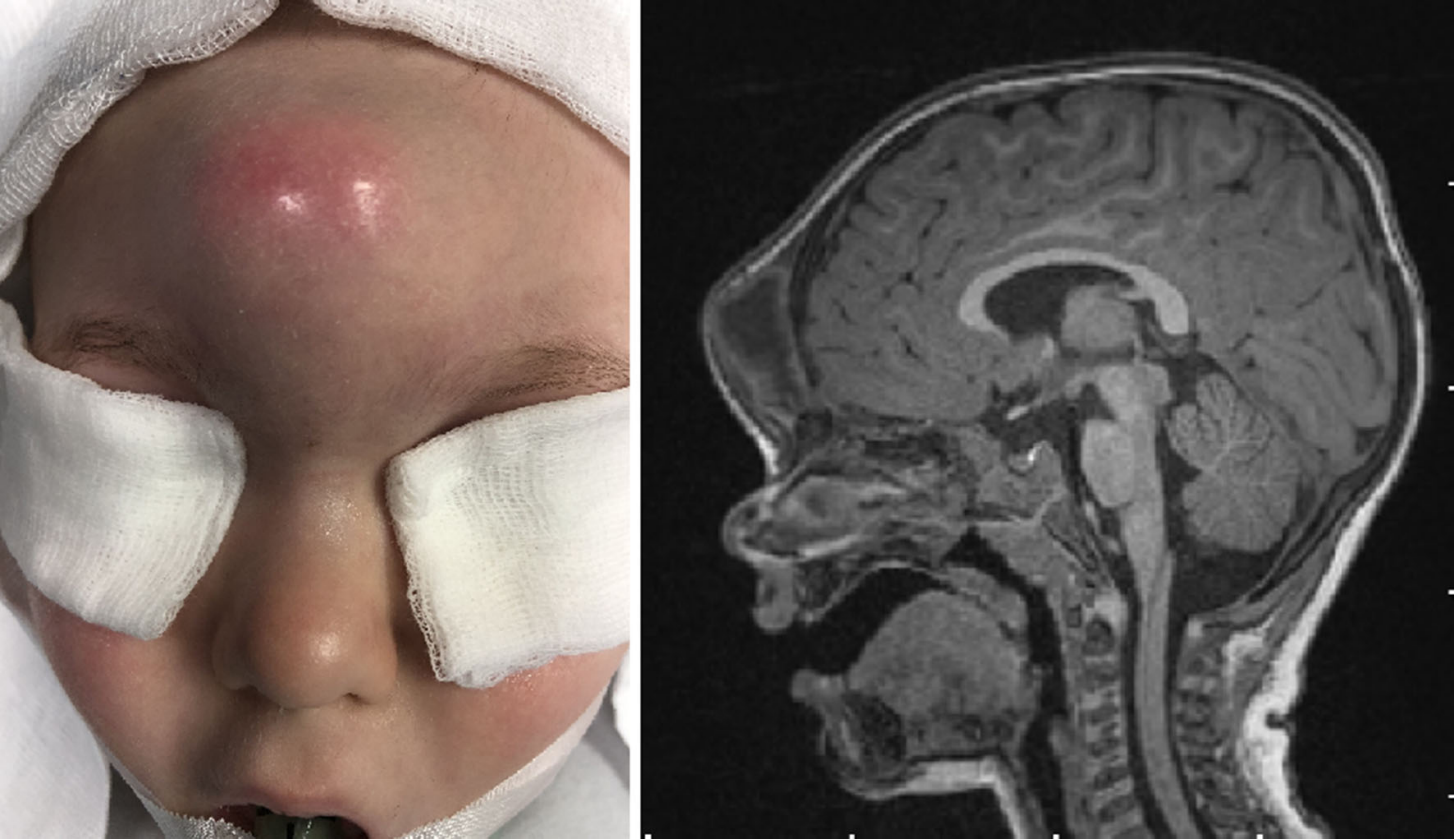
Complication of the nasal dermoid in 18-month-old male patient. A massive abscess of the frontal region—clinical manifestation and MRI-T1 sagittal view

The analysis of recent years’ publications indicates the growing consensus on the proper age for surgery. Most authors advocate to operate as early as possible, even during infancy [10, 14, 17, 18]. The aim is to minimize the risk of infectious complications and prevent bony remodeling [10, 18]. A distortion of the nasal bones and their atrophy and cartilage deformation resulting from the mass expansion make the surgical procedure more demanding with an unpredictable distant cosmetic result. The factors influencing the latter haven’t been analyzed and published so far. In our group, 33% of patients reported recurrent episodes of local inflammation whereas in 6 out of 24 cases the significant deformation of the nose was visible. The analysis of demographic data of these patients shed a new light on the issue. Seven out of 8 cases with preoperative local inflammations and 3 out of 4 with secondary fistulization were < 4 years old. We conclude that the incidence of local inflammatory complications is unrelated to the age of the patients. The older children did not present the inflammatory complications more often. In one particular case, the massive abscess in the frontal region was observed, and the enormous deformity of the frontal bone was revealed intraoperatively (Fig. 2). No episodes of meningitis were observed in the study group. Similarly, it was not reported in the largest patients’ series [10, 12, 16].

### Presurgical Considerations and Radiological Imaging

Paller et al. claimed that columellar localization and the isolated cyst for were associated with a lower risk of intracranial involvement [23]. Danoyelle et al. reported that 5 out of 6 patients with intracranial extension in their series presented a sinus ostium, but only 19% of 26 children with sinus ostium had intracranial extension [10]. Our study revealed no correlation between intracranial extension and the sinus ostium or cyst location, similar to the reports by Rahbar et al. and Bradley et al. [12, 13]. Among 8 children with intracranial involvement, one child presented a cyst and another 1 a sinus of the glabellar region, 5 patients manifested a sinus ostium on the nasal bridge, and 1 presented a philtrum sinus. No columellar locations were observed.

**Fig. 3:**
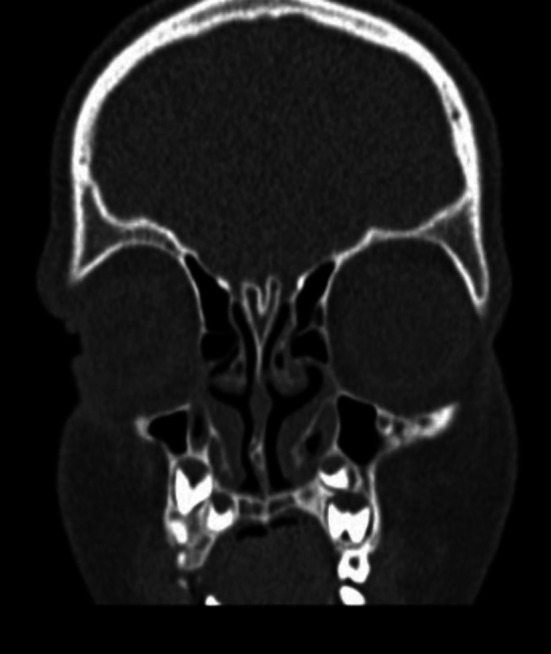
Bifid crista galli—CT coronal scan

The lack of correlation between the position of a superficial cyst or skin ostium of the sinus strengthens the role of obligatory preoperative radiological imaging. All children with nasal dermoid are suspected of intracranial extension, but there is an ongoing debate in the literature concerning the most suitable radiological tool. Posnick et al. suggested CT scanning for all patients before the surgery [24]. There are characteristic radiological signs of possible intracranial extension on CT scans, an isolated widening of the foramen caecum and bifid crista galli [5, 10, 24] (Fig. 3). According to Bloom et al., they are only suggestive, not diagnostic for intracranial extension [18]. However, if these criteria are absent—they eliminate the presence of intracranial extension [5].

There are opinions that MRI alone is sufficient for precise evaluation of intracranial extension [18, 25]. Bloom et al. recommend MRI as initial imaging for all patients because of the risk of a second general anesthetic and the additional imaging cost [18]. Some researchers emphasize that all cases of positive or indeterminate CT scan require an MRI [10, 18] to eliminate false-positive CT scan studies [5, 7, 23] and avoid needless intracranial approach [5, 10, 18].

Our radiological analysis revealed 3 false-negative MRI results, 1 false-negative CT and 2 false-positive CT scans in our patients. As MRI technique characterizes with higher predictive values in terms of identifying intracranial extension of nasal dermoid on imaging, it should become a diagnostic tool of choice [26]. Barkovich et al. suggested evaluating all the patients with both CT and MR imaging [27] which seems to be overdiagnosis. In order to reduce the incidence of false-positive and false-negative results concerning intracranial extension, the combination of CT and MRI is highly recommended only for children below the age of 5 [26]. Awareness of the most common radiological pitfalls and natural developmental processes of the anterior skull base support our conclusions [5, 10, 18, 24, 28–30].

### Surgical Considerations

There is a wide variety of proposed surgical approaches: medial excision and direct closure, external rhinoplasty, bicoronal excision with or without craniotomy, small window craniotomy, etc. The majority of lesions are successfully removed using vertical incision [12, 19] or external rhinoplasty approach [10]. Some authors claim that open rhinoplasty has some benefits over direct incision: more predictable esthetic result, better visualization of the cribriform plate or wider exposure with better control over osteotomies [30]. Other researchers stress that medial vertical incision with direct excision provides adequate exposure, excellent direct visualization, bimanual dissection, limited dissection of the nasal complex, and favorable cosmetic result [19]. Nevertheless, the external rhinoplasty’s advantages become disputable in very young patients [31] or cases of deep intraosseous lesions, especially with intracranial extension. Similarly, in children with additional sinus ostium or secondary fistulization, their benefit diminishes as they have to be accompanied by additional external approaches. It stays contrary to Danoyelle et al. and Bloom et al., who states that the earlier the surgery the easier external rhinoplasty is [10, 18].

Bradley et al. reported that 54% of their patients were treated with direct excision and closure and almost 36% via an external rhinoplasty approach but did not precisely present their cosmetic results [12]. In the studies of Danoyelle et al., 64% of lesions were removed by external rhinoplasty, whereas 19.5% by midline vertical incision. In 88.9% of children, the aesthetic results were judged as satisfactory [10]. Cheng& Kazahaya prefer direct excision even in patients with intracranial extension as they operated on a series of 20 pediatric patients with good esthetic results [19]. In our study group, 87, 5% of patients were treated with midline vertical excision, 8.3% of children with external rhinoplasty, and 1 case required a bicoronal approach. The distant cosmetic result assessment revealed that in 29% it was regarded as satisfactory, good in 46%, and excellent in 12.5% of patients. All the patients from the first subgroup had the positive history of recurrent local inflammations or a variety of topical surgical interventions. As the mean age was comparable in these 3 subgroups, we conclude that the distant aesthetic effect depends rather on both the initial extent of the disease and preoperative local conditions or interventions, than solely the age of the patient. The issue requires further studies on large groups of patients.

We state by the view that lateral and intermediate osteotomies become necessary in two particular clinical situations: to provide adequate access to the deep intraosseous lesion and to close the open roof in case of partial nasal bone destruction. Among 13 patients presenting an intraosseous or intracranial form of abnormality in our study group, osteotomies were performed in 9 children (69%).

Cases requiring highly individual surgical planning are patients with skin damage due to secondary fistulization, with multilocular lesions, or these presenting nasal bone destruction. In our cohort of patients, 4 children presented nasolateral fistulization with granulation tissue proliferation secondary to the cystic part of the abnormality's recurrent inflammation. Three of them required additional horizontal incision and one — patracanthal approach. In one case, there was a massive abscess formation in the frontal region treated by bicoronal approach combined with direct excision of the glabellar sinus ostium.

**Fig. 4:**
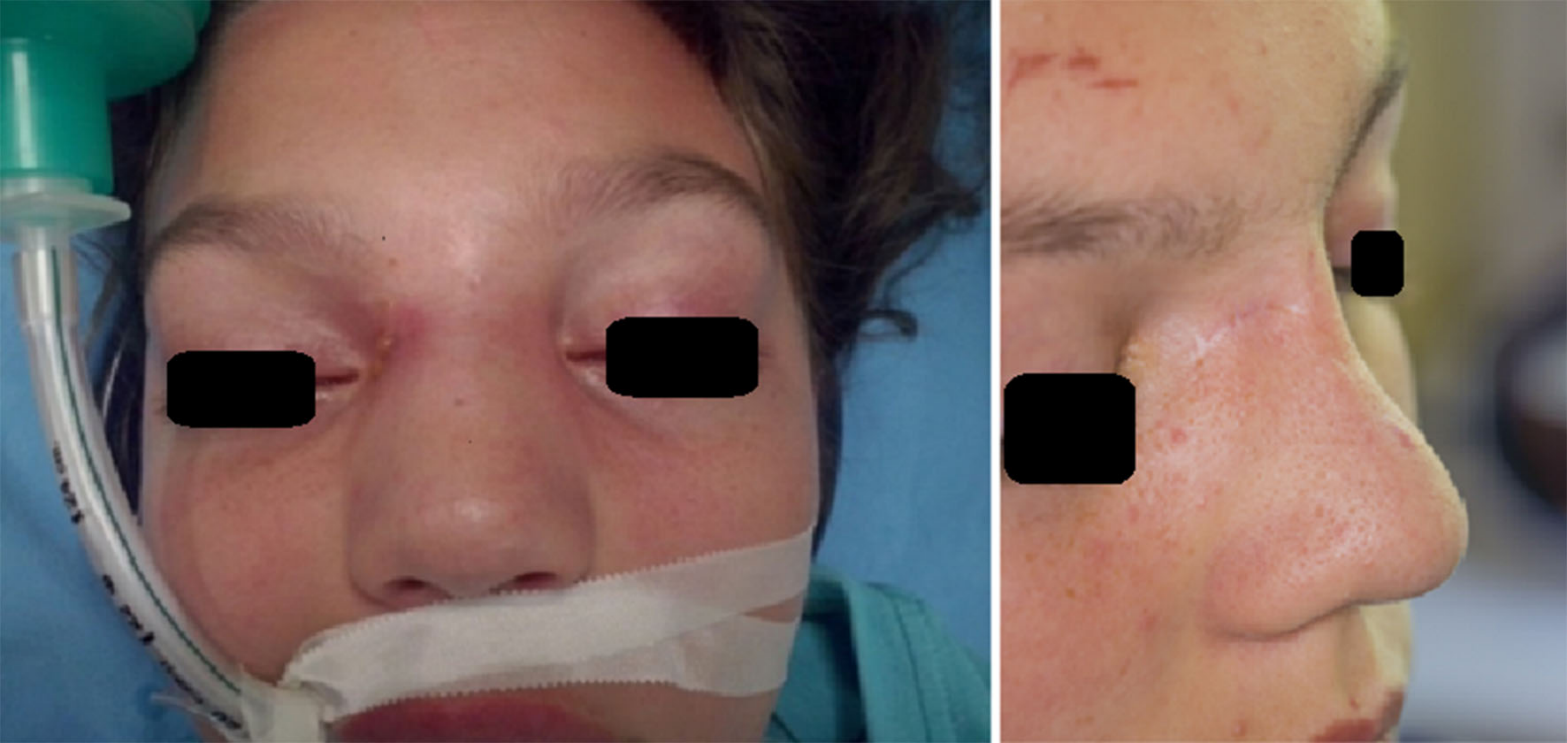
Reconstruction of the nasal skeleton with the cartilage costal graft–the preoperative view and the distant cosmetic result

There is a scarce of reports concerning the influence of nasal bone destruction in NDs on further facial skeleton development. Basing on the experience of 3 out of 24 of our patients, we suggest a simultaneous reconstruction. In one case of partial nasal bone destruction, a fat graft was implemented. The second patients’ deformity of the frontal bone and glabellar region resulting from the cyst and abscess of that area was reconstructed with bony chips. The subtotal unilateral nasal bone destruction, causing the the nasal bridge’s deformity, was revealed in the third patient. The correction with cartilage costal graft was performed (Fig. 4). A similar technique was proposed by Mankarious & Smith [32]. Being aware of the potential necessity of further rhinoplasty after puberty, an informed consent concerning the issue should be attached to medical documentation.

Cerebrospinal fluid leak is a potential intraoperative complication of dissection when the dural attachment of the lesion presents a tight adhesion. We experienced it twice, and it was simultaneously typically repaired without any further consequences. Although CSF leak is considered a serious complication, it is beneficial to avoid craniotomy and its disadvantages in intracranial extradural dermoids.

## Conclusion

The local inflammatory complications of NDs are unpredictable and their incidence is unrelated to the age of the patients. The distant aesthetic result depends rather on both the initial extent of the disease and preoperative local conditions or interventions, than solely the age of the patient. Prompt surgical intervention is highly recommended. The wide variety of surgical solutions is expected intraoperatively due to the preoperative radiological studies' limitations.

## Data Availability

The data that support the findings of this study are available on request from the corresponding author. The data are not publicly available due to restrictions (the privacy of research participants).
